# Association Between Dietary Patterns and Sarcopenia in Patients With Liver Cirrhosis: A Cross‐Sectional Study

**DOI:** 10.1002/hsr2.72610

**Published:** 2026-06-11

**Authors:** Elham Sobhrakhshankhah, Parvin Hassanzadeh, Soghra Karimi, Mohsen Reza Mansoorian, Naemeh Kolahdoozan, Azam Doustmohammadian

**Affiliations:** ^1^ Gastrointestinal and Liver Diseases Research Center Iran University of Medical Sciences Tehran Iran; ^2^ Department of Internal Medicine, School of Medicine, Firoozgar General Hospital Iran University of Medical Sciences Tehran Iran

**Keywords:** dietary patterns, liver cirrhosis, nutritional assessment, principal component analysis, sarcopenia

## Abstract

**Background and Aims:**

Sarcopenia is a prevalent and debilitating complication in patients with liver cirrhosis, contributing to poor clinical outcomes. Although diet quality is believed to influence muscle health, the role of overall dietary patterns in patients with cirrhosis remains inadequately explored. This study examined the relationship between dietary patterns and sarcopenia in individuals with liver cirrhosis.

**Methods:**

In this cross‐sectional study, 92 adults with clinically confirmed liver cirrhosis were enrolled from a tertiary academic center between February 2024 and January 2025. Standardized protocols were used to collect sociodemographic data, anthropometric measurements, physical activity data, and dietary intake information. Principal component analysis identified major dietary patterns based on a validated 168‐item food frequency questionnaire. Sarcopenia was diagnosed using AWGS 2019 criteria, incorporating mid‐upper arm circumference, handgrip strength, and the 6‐min walk test. Multivariable logistic regression models examined associations between dietary patterns and sarcopenia, adjusting for age, sex, energy intake, physical activity, and comorbidities.

**Results:**

Sarcopenia was identified in 66.3% of participants. Patients with sarcopenia exhibited significantly lower BMI, mid‐arm and calf circumference, and physical performance compared to their non‐sarcopenic counterparts (all *p* < 0.01). Adherence to a Western dietary pattern, characterized by high intake of red and processed meats, refined grains, and fried foods, was positively associated with sarcopenia (adjusted OR for second tertile: 5.99; 95% CI: 1.53–23.43). Conversely, higher adherence to a healthy dietary pattern, rich in vegetables, nuts, oils, and whole grains, was inversely associated with sarcopenia (adjusted OR for the highest tertile: 0.35; 95% CI: 0.11–1.52).

**Conclusion:**

Our findings highlight the potential of dietary pattern modification as a strategy for preventing sarcopenia in cirrhotic patients. Integrating nutritional screening and dietary counseling into routine hepatology care may offer a low‐risk, cost‐effective approach to improving functional outcomes in this high‐risk population.

## 1 Introduction

Liver cirrhosis (LC) represents the final, irreversible stage of liver damage and fibrosis and is associated with substantial morbidity and mortality worldwide [1]. As the prevalence of liver disease continues to rise, particularly due to metabolic dysfunction‐associated steatotic liver disease (MASLD), the clinical management of cirrhosis remains a major public health challenge [2]. Among the most common and debilitating complications of cirrhosis is sarcopenia, defined by the progressive loss of skeletal muscle mass, strength, and physical performance, which affects up to 70% of patients and has been independently linked to poor clinical outcomes, including increased hospitalization, reduced quality of life, and higher mortality rates [3, 4].

While the etiology of sarcopenia in cirrhosis is multifactorial, encompassing chronic inflammation, hormonal imbalances, physical inactivity, and malnutrition, nutritional factors are increasingly recognized as modifiable contributors to muscle decline [5, 6]. Historically, studies have focused on individual nutrients or caloric intake [7]; however, growing evidence suggests that overall dietary patterns, which reflect habitual food choices and nutrient interactions, may better capture the complexity of diet–disease relationships [8]. In particular, dietary patterns rich in anti‐inflammatory and antioxidant‐rich foods have been shown to support muscle health, while Western‐style diets high in saturated fats and ultra‐processed foods may exacerbate systemic inflammation and muscle catabolism [9, 10, 11].

Despite these insights, data on the association between dietary patterns and sarcopenia in patients with cirrhosis remain scarce [7]. Most existing research has emphasized clinical and biochemical predictors, with limited attention to comprehensive dietary assessment [12]. Understanding the role of habitual diet in the development or prevention of sarcopenia may inform targeted interventions that are both non‐invasive and cost‐effective.

Accordingly, this study explored the relationship between data‐driven dietary patterns and the presence of sarcopenia in adults diagnosed with liver cirrhosis. By identifying dietary patterns associated with increased or reduced risk of sarcopenia, our findings may provide a basis for integrating nutritional strategies into the routine care of patients with liver disease.

## 2 Methods

### 2.1 Study Design and Setting

This cross‐sectional study was conducted between February 2024 and January 2025 at Firouzgar Hospital, a leading tertiary care and academic referral center affiliated with Iran University of Medical Sciences in Tehran. The investigation focused on adult patients (≥ 18 years) with clinically confirmed cirrhosis of varying etiologies and severity, recruited from both the hospital's Liver Transplantation Center and outpatient hepatology clinic.

### 2.2 Study Population and Sampling Method

Participants were consecutively enrolled among eligible patients referred during the study period. Inclusion criteria required a definitive diagnosis of cirrhosis established by a composite of clinical history, physical examination, biochemical markers, imaging findings, or liver biopsy, where indicated. All participants provided informed written consent and demonstrated the capacity to comply with study procedures.

Patients were excluded if they had severe systemic comorbidities likely to confound study outcomes, including but not limited to chronic obstructive pulmonary disease (COPD), chronic kidney disease, heart failure, or active malignancy. Individuals unable to provide informed consent were also excluded.

Sample size determination was guided by prior data on handgrip strength in cirrhotic patients, which reported a standard deviation (σ) of 3.52 kg and a mean of 16.9 kg [13]. Using a precision level (d) of 1, a significance level (α) of 0.01, and a two‐tailed *Z*‐score, the minimum required sample size was calculated as 82. To ensure adequate statistical power and account for potential attrition, a total of 100 participants were ultimately enrolled.

### 2.3 Data Collection and Clinical Assessment

Each participant completed a comprehensive, interviewer‐administered questionnaire that captured sociodemographic characteristics (age, sex, education, occupation, marital status, and income), medical history (comorbidities, smoking status, etiology of cirrhosis, and medication use), and disease severity.

### 2.4 Anthropometric Measurements

Anthropometric measurements were conducted following standardized procedures. Body weight was assessed to the nearest 0.1 kg using a calibrated digital scale (Seca, Germany), with participants wearing light clothing and no footwear. Standing height was recorded with a Seca stadiometer to the nearest 0.1 cm. Waist circumference was measured midway between the lowest rib and the iliac crest at the end of a normal exhalation, while hip circumference was taken at the widest part over the greater trochanters. Abdominal obesity was defined as a waist circumference greater than 102 cm for males and 88 cm for females, according to NCEP‐ATP III sex‐specific cutoffs [14, 15]. Calf circumference was measured at the widest point of the calf while the participant was standing upright with feet 20 cm apart, using a non‐stretchable measuring tape, as recommended by the WHO STEPS protocol [16]. Low calf circumference was defined as < 34 cm for men and < 33 cm for women, based on a study on Iranian adults and AWGS criteria [17]. Mid‐upper arm circumference (MUAC) was measured on the non‐dominant side at the midpoint between the acromion and olecranon, with the elbow bent at a 90° angle, and recorded to the nearest 0.1 cm.

### 2.5 Sarcopenia Diagnosis

Sarcopenia was identified based on the 2019 criteria of the Asian Working Group for Sarcopenia (AWGS), characterized by reduced muscle mass in conjunction with diminished muscle strength and/or low muscle function [17]. Severe sarcopenia involves decreased muscle strength, mass, and physical performance.

Skeletal muscle mass was assessed using mid‐upper arm circumference (MUAC), measured with a non‐elastic tape to the nearest 0.1 cm. Based on EWGSOP2 recommendations, low muscle mass was defined as MUAC ≤ 28 cm for males and ≤ 27 cm for females [18].

Handgrip strength (HGS) was measured using a calibrated Jamar dynamometer (Model 14192‐709E) in the dominant hand. Each participant performed three consecutive handgrip tests, with 30‐second intervals between attempts. The highest value was recorded, and the mean maximum strength was used to measure muscle strength. Low handgrip strength was classified as less than 27 kg in males and below 16 kg in females [19].

Physical performance was evaluated using the 6‐Minute Walk Test (6MWT) conducted along a 30‐m corridor, in accordance with international guidelines [20]. The distance covered in 6 min was recorded, and a distance of less than 250 m indicated poor physical performance [21].

### 2.6 Physical Activity Assessment

Physical activity levels were assessed using the validated short form of the International Physical Activity Questionnaire (IPAQ‐SF) [22]. Participants reported the frequency and duration of various physical activities across four domains, including work, transportation, household, and leisure, during the previous week. Total physical activity was calculated in metabolic equivalent (MET) minutes per week and initially categorized as low (< 600 MET‐min/week), moderate (600–3000 MET‐min/week), or high (> 3000 MET‐min/week). Given the low proportion of participants in the high physical activity category (2.2%), we combined the moderate and high categories into a single moderate‐to‐high physical activity group for subsequent analyses.

### 2.7 Nutritional Assessment

Habitual dietary intake over the preceding year was assessed via a reliable and validated semi‐quantitative 168‐item Food Frequency Questionnaire (FFQ) [23]. For each food item included in the questionnaire, participants indicated their habitual frequency of consumption, expressed in standard portion sizes on a daily, weekly, or monthly basis, reflecting their dietary patterns over the preceding 12 months. The mean daily intake for each item was subsequently estimated in grams per day using customary household measures [24]. Nutrient and energy contents were quantified using the Food Composition Table of the United States Department of Agriculture (USDA) [25] and the Iranian Food Composition Table, which encompasses data for traditional Iranian foods [26].

### 2.8 Preliminary Assessment of Dietary Patterns

To identify underlying dietary patterns, we conducted an exploratory factor analysis (EFA) on 23 predefined food groups. These food items, derived from the FFQ, were organized into the categories based on similarities in nutrient composition and the availability of supporting data [27, 28, 29, 30], providing the framework for the factor analysis. Table S1 indicates the components of food groups included in dietary patterns (Table S1). Factor scores for each identified dietary pattern were derived by summing the standardized intake of associated food groups, weighted according to their factor loadings. Only food groups with loadings exceeding 0.20 were considered [31], reflecting the strongest contributors to each pattern. Patterns were subsequently labeled based on the highest loadings and conceptual interpretability. Participants were then stratified into tertiles based on their factor scores to facilitate further analysis.

### 2.9 Ethical Considerations

This study adhered to the ethical principles outlined in the Declaration of Helsinki. Ethical approval was granted by the Ethics Committee of the Iran University of Medical Sciences (IUMS) under reference number IR.IUMS.FMD.REC.1404.218. Written informed consent was obtained from all individuals before participation.

### 2.10 Statistical Analysis

**Figure 1:**
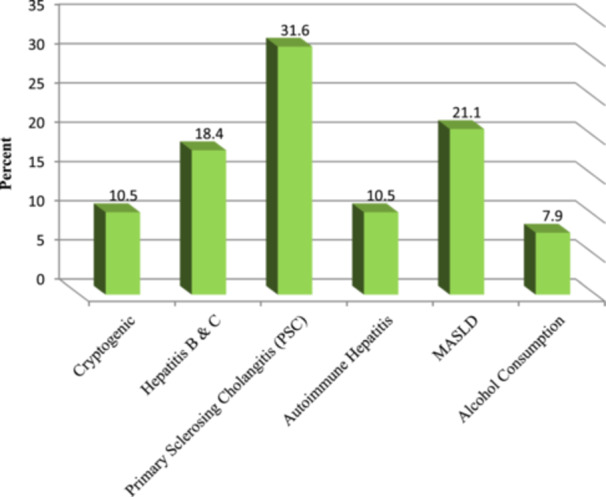
Distribution of cirrhosis etiologies among patients. Metabolic dysfunction‐associated steatotic liver disease (MASLD).

Statistical analyses were conducted using RStudio (version 2024.12.1‐563) and IBM SPSS Statistics (version 26). Continuous variables were summarized as means with standard deviations (SD) or medians with interquartile ranges (IQR), while categorical data were presented as frequencies and percentages. The Kolmogorov–Smirnov test was applied to assess the normality of data distributions. The primary dietary patterns were identified through Principal Component Analysis (PCA), followed by orthogonal varimax rotation to enhance interpretability [32, 33]. The optimal number of factors retained was determined using a combination of criteria, including eigenvalues exceeding 1.5, visual inspection of the scree plot (Figure 1), and the conceptual clarity of the extracted components [34]. The suitability of the data set for factor analysis was evaluated using the Kaiser–Meyer–Olkin (KMO) measure of sampling adequacy and Bartlett's test of sphericity. KMO values were 0.60, while Bartlett's test indicated significant sphericity (*p* < 0.001), confirming the appropriateness of the data for further analysis. Factor scores were computed for each participant, reflecting adherence to identified dietary patterns.

Group comparisons were conducted using either independent *t*‐tests or Mann–Whitney U tests, while categorical variables were assessed using *χ* ^2^ or Fisher's exact tests. Analysis of variance (ANOVA) with Bonferroni post hoc correction was employed for multi‐group comparisons.

To evaluate associations between dietary patterns (independent variables) and sarcopenia, logistic regression models were constructed. Model 1 was adjusted for age, sex, and total energy intake. Model 2 included additional covariates: physical activity level, waist circumference, alcohol consumption, and presence of comorbidities. A crude (unadjusted) model was also reported. Statistical significance was defined as a two‐tailed *p* value ≤ 0.05.

## 3 Results

### 3.1 General Characteristics of the Study Population

A total of 92 patients with liver cirrhosis were included in the final analysis (Table 1). The mean age of the participants was 49.10 ± 13.45 years, and 57.6% were male. Based on diagnostic criteria, 33.7% of participants were categorized as having sarcopenia, and 32.6% as having severe sarcopenia. For analytical purposes, patients with sarcopenia and severe sarcopenia were combined into a single group (*n* = 61, 66.3%), and those with pre‐sarcopenia or no sarcopenia formed the reference group (*n* = 31, 33.7%).

**Table 1:** Demographics and clinical characteristics of all patients.

Variable	Total (*N* = 92)	Non‐sarcopenia group (*n* = 31)	Sarcopenia group (*n* = 61)	*p* value*
Age (years)	49.10 ± 13.45	50.45 ± 13.12	48.41 ± 13.67	0.494
Gender, *n* (%)				
Male	53 (57.6)	22 (71.0)	31 (50.8)	0.065
Female	39 (42.4)	9 (29.0)	30 (49.2)	
Occupation, *n* (%)				
Unemployed	27 (29.3)	7 (22.6)	20 (32.8)	0.595
Employee/retired	22 (23.9)	8 (25.8)	14 (23.0)	
Self‐employed	43 (46.7)	16 (51.6)	27 (44.3)	
Economic status, *n* (%)				
Poor	38 (41.3)	10 (32.3)	28 (45.9)	0.422
Moderate	45 (48.9)	18 (58.1)	27 (44.3)	
Good	9 (9.8)	3 (9.7)	6 (9.8)	
Marital status, *n* (%)				
Single/divorced/widowed	20 (21.7)	3 (9.7)	17 (27.9)	**0.046**
Married	72 (78.3)	28 (90.3)	44 (72.1)	
Education level, *n* (%)				
Illiterate	12 (13.0)	3 (9.7)	9 (14.8)	0.313
High school diploma or lower	65 (70.7)	25 (80.6)	40 (65.6)	
University degree	15 (16.3)	3 (9.7)	12 (19.7)	
Physical activity (MET)^a^, *n* (%)				
Low	72 (78.3)	24 (77.4)	48 (78.7)	0.889
Moderate to high	20 (21.7)	7 (22.6)	13 (21.3)	
Anthropometric indices				
Weight (kg)	72.29 ± 15.02	79.47 ± 13.32	68.65 ± 14.61	**0.001**
Height (cm)	169.47 ± 13.22	168.24 ± 16.56	170.10 ± 11.27	0.528
Body mass index (BMI)	24.81 ± 3.59	27.12 ± 3.03	23.64 ± 3.86	**< 0.001**
Waist circumference (cm)	92.86 ± 12.10	97.93 ± 10.64	90.29 ± 12.06	**0.004**
Arm circumference (cm)	27.78 ± 3.76	29.89 ± 2.70	26.70 ± 3.79	**< 0.001**
Calf circumference (cm)	35.49 ± 5.99	38.56 ± 7.03	33.93 ± 4.73	**< 0.001**
Abdominal obesity^b^				
No	49 (53.3)	13 (41.9)	36 (59.0)	0.092
Yes	43 (46.7)	18 (58.1)	25 (41.0)	
Time since the diagnosis of cirrhosis, (month), Median (IQR)	16.50 (43.0)	12 (31.0)	24 (49.0)	0.526
Child–Pugh classification, *n* (%)				
A + B	85 (92.4)	27 (31.8)	58 (95.1)	0.220
C	7 (7.6)	4 (12.9)	3 (4.9)	
Hospitalization				
No	15 (16.3)	6 (19.4)	9 (14.8)	0.572
Yes	77 (83.7)	25 (80.6)	52 (85.2)	
Presence of comorbidities, *n* (%)				
No	59 (64.1)	17 (54.8)	42 (68.9)	0.185
Yes	33 (35.9)	14 (45.2)	19 (31.1)	
Low muscle strength^c^, ^d^, *n* (%)	34 (37.0)	15 (48.4)	19 (31.1)	0.105
Low muscle function^c^, ^d^, *n* (%)	80 (87.0)	20 (64.5)	60 (98.4)	**< 0.001**
Low muscle mass^e^, *n* (%)	48 (52.2)	7 (22.6)	41 (67.2)	**< 0.001**

^a^ Low levels of physical activity (PA): (PA) over the past 7 days < 600 (Met‐min/week), moderate levels of (PA): (600–3000 MET‐ min/week) and high levels of (PA): (> 3000 MET‐min/week) due to the low percentage (2.2%) in the high levels of (PA), we combined the two levels of moderate and high to moderate to high (PA).

^b^ Waist circumference ≥ 102 cm for men and ≥ 88 cm for women was defined as abdominal obesity based on NCEP‐ATP III criteria.

^c^ Low muscle strength < 27 kg for females and for males, muscle strength < 16 kg for females.

^d^ A 6MWD of less than 250 m represents low muscle function.

^e^ Low muscle mass: upper arm circumference < 27.5 cm for females and for males upper arm circumference < 28.6 cm.

* Bolded values are significant at the < 0.05 level.

There were no significant differences in age or gender between the sarcopenic and non‐sarcopenic groups. However, marital status was significantly associated with sarcopenia (*p* = 0.046), with a higher proportion of single, divorced, or widowed individuals observed in the sarcopenia group (27.9%) compared to the non‐sarcopenia group (9.7%).

Anthropometric indicators, including weight, BMI, waist circumference, arm circumference, and calf circumference, were significantly lower in individuals with sarcopenia. For instance, mean BMI in the sarcopenia group was 23.64 compared to 27.12 in the non‐sarcopenia group (*p* < 0.001). Similarly, calf circumference was markedly reduced in the sarcopenia group (33.93 cm vs. 38.56 cm; *p* < 0.001). No significant association was observed between abdominal obesity and sarcopenia status. Although a higher proportion of individuals without sarcopenia had abdominal obesity (58.1%) compared to those with sarcopenia (41.0%), the difference did not reach statistical significance (*p* = 0.092).

Muscle‐related parameters, including strength and function, followed a similar pattern: 98.4% of sarcopenic individuals had impaired muscle function compared to 64.5% of those without sarcopenia (*p *< 0.001). Additionally, low muscle mass was more prevalent in the sarcopenia group (67.2% vs. 22.6%; *p*< 0.001). Primary sclerosing cholangitis and Metabolic dysfunction‐associated steatotic liver disease (MASLD) were the main causes of hospitalization (31.6% and 21.1%, respectively), followed by viral hepatitis B/C (18.4%) (Figure 1).

### 3.2 Identified Dietary Patterns

Principal Component Analysis identified four distinct dietary patterns: Western, Unhealthy, Healthy, and Plant‐Based and Light. The Western pattern was characterized by high intakes of red and processed meats, organ meats, fried foods, and refined grains. The Unhealthy pattern included high salt, condiments, full‐fat dairy, and processed items. The Healthy pattern was defined by higher consumption of vegetables, nuts, liquid oils, and whole grains. The Plant‐Based and Light pattern emphasized legumes, tea, spices, and boiled potatoes, with lower intake of animal products. This analysis is illustrated in Table 2 and Figure S1.

**Table 2:** Factor loadings for dietary patterns extracted from factor analysis^a^.

Food groups	Western dietary pattern	Unhealthy dietary pattern	Healthy dietary pattern	Plant‐based and light dietary pattern
Organ meats	**0.882**	0.064	0.034	−0.079
Refined grains	**0.797**	0.153	−0.055	0.169
Red meat	**0.747**	0.233	−0.104	−0.315
Processed meat	**0.497**	0.419	−0.005	−0.100
Fried potatoes	**0.484**	0.260	0.017	0.025
Sweetened beverages	**0.350**	0.348	0.070	0.232
Eggs	**0.213**	−0.119	−0.005	0.111
Condiments	0.188	**0.912**	0.009	0.023
High fat dairy	0.192	**0.714**	0.104	−0.081
Salt	−0.004	**0.622**	0.062	0.281
Low fat dairy	−0.055	**0.495**	−0.063	−0.108
Solid fats	0.152	**0.349**	−0.199	−0.070
Salty sweet snacks	0.126	**0.289**	−0.006	0.186
Vegetables	−0.107	−0.030	**0.805**	−0.183
Liquid oils	−0.088	−0.068	**0.754**	0.211
Whole grains	0.394	−0.185	**0.740**	−0.081
Nuts	−0.034	0.214	**0.686**	−0.206
Fruits	−0.050	0.052	**0.145**	−0.565
White meats	−0.051	−0.017	**0.313**	−0.559
Legumes	0.121	−0.049	0.078	**0.500**
Tea and coffee	−0.012	0.229	0.026	**0.436**
Cooked potatoes	−0.174	−0.060	−0.048	**0.351**
Spices	−0.160	0.111	0.112	**0.203**

^a^ Values are factor loadings showing the strength and direction of association between food groups and dietary patterns. Factor loadings > 0.2 are in bold font.

Analysis across dietary pattern tertiles revealed significant associations between diet and sarcopenia‐related variables (Tables 3 and 4). Participants in the second tertile of the Western dietary pattern had a significantly higher prevalence of sarcopenia (83.9%) compared to those in the first tertile (53.3%; *p* = 0.032). Similar trends were observed for muscle function impairment (100% vs. 80%; *p* = 0.030). For the Healthy dietary pattern, sarcopenia was significantly more prevalent in the lowest tertile (70%) compared to the highest (45.2%; *p* = 0.005), suggesting a potential protective effect of this pattern.

**Table 3:** Baseline characteristics of the study participants across categories of dietary pattern scores.

Dietary patterns	Western dietary pattern			*p* value	Unhealthy dietary pattern			*p* value*
	T1 (*n* = 30)	T2 (*n* = 31)	T3 (*n* = 31)		T1 (*n* = 30)	T2 (*n* = 31)	T3 (*n* = 31)	
Age (years)	52.17 ± 10.90	46.87 ± 15.37	48.35 ± 13.50	0.289	72.29 ± 15.02	79.47 ± 13.32	68.65 ± 14.61	**0.012**
Gender, *n* (%)								
Male	13 (43.3)	19 (61.3)	21 (67.7)	0.137	19 (63.3)	17 (54.8)	17 (54.8)	0.776
Female	17 (56.7)	12 (38.7)	10 (32.3)		11 (36.7)	14 (45.2)	14 (45.2)	
Occupation, *n* (%)								
Unemployed	12 (40.0)	9 (29.0)	6 (19.4)	0.494	9 (30.0)	8 (25.8)	10 (32.3)	0.941
Employee/retired	7 (23.3)	7 (22.6)	8 (25.8)		7 (23.3)	9 (29.0)	6 (19.4)	
Self‐employed	11 (36.7)	15 (48.4)	17 (54.8)		14 (46.7)	14 (45.2)	15 (48.4)	
Economic status, *n* (%)								
Poor	12 (40.0)	15 (48.4)	11 (35.5)	0.796	10 (33.3)	17 (54.8)	11 (35.5)	0.145
Moderate	16 (53.3)	13 (41.9)	16 (51.6)		19 (63.3)	11 (35.5)	15 (48.4)	
Good	2 (6.7)	3 (9.7)	4 (12.9)		1 (3.3)	3 (9.7)	5 (16.1)	
Marital status, *n* (%)								
Single/divorced/widowed	2 (6.7)	11 (35.5)	7 (22.6)	**0.024**	3 (10.0)	13 (41.9)	4 (12.9)	**0.004**
Married	28 (93.3)	20 (64.5)	24 (77.4)		27 (90.0)	18 (58.1)	27 (87.1)	
Education status, *n* (%)								
Illiterate	3 (10.0)	7 (22.6)	2 (6.5)	0.203	4 (13.3)	5 (16.1)	3 (9.7)	0.870
High school diploma or lower	20 (66.7)	19 (61.3)	26 (83.9)		20(66.7)	21 (67.7)	24 (77.4)	
University degree	7 (23.3)	5 (16.1)	3 (9.7)		6 (20.0)	5 (16.1)	4 (12.9)	
Physical activity^a^ (MET)								
Low PA	23 (76.7)	26 (83.9)	23 (74.2)	0.631	24 (80.0)	23 (74.2)	25 (80.6)	0.856
Moderate to high PA	7 (23.3)	5 (16.1)	8 (25.8)		6 (20.0)	8 (25.8)	6 (19.4)	
Anthropometric indices								
Weight (kg)	74.43 ± 16.63	71.77 ± 13.93	70.75 ± 14.70	0.619	75.02 ± 14.62	68.78 ± 14.34	73.17 ± 15.86	0.251
Height (cm)	164.75 ± 15.75	172.40 ± 12.56	171.11 ± 9.97	0.053	168.95 ± 12.17	171.32 ± 11.20	168.13 ± 16.01	0.620
Body mass index (BMI)	26.20 ± 3.46	24.21 ± 4.33	24.07 ± 3.77	0.062	26.27 ± 3.84	23.37 ± 3.57	24.85 ± 4.02	**0.015**
Waist circumference (cm)	95.35 ± 12.94	92.32 ± 11.34	91.02 ± 11.99	0.363	95.95 ± 13.14	90.64 ± 10.08	92.11 ± 12.67	0.213
Arm circumference (cm)	28.78 ± 3.65	27.17 ± 4.09	27.40 ± 3.43	0.2	28.37 ± 3.60	26.84 ± 3.88	28.14 ± 3.73	0.230
Calf circumference (cm)	36.95 ± 7.67	34.46 ± 5.75	35.11 ± 3.94	0.247	35.88 ± 3.64	35.13 ± 4.19	35.13 ± 4.20	0.888
Etiology of cirrhosis, *n* (%)								
Alcoholic cirrhosis	1 (3.3)	2 (6.5)	6 (19.4)	0.509	2 (6.7)	1 (3.2)	6 (19.4)	**0.031**
Hepatitis	1 (3.3)	2 (6.5)	1 (3.2)		0 (0.0)	1 (3.2)	3 (9.7)	
Alcohol consumption	2 (6.7)	0 (0.0)	1 (3.2)		0 (0.0)	0 (0.0)	3 (9.7)	
Hepatitis B and C	1 (3.3)	4 (12.9)	2 (6.5)		1 (3.3)	3 (9.7)	3 (9.7)	
Autoimmune hepatitis	5 (16.7)	3 (9.7)	6 (19.4)		7 (23.7)	3 (9.7)	4 (12.9)	
Primary sclerosing cholangitis (PSC)	5 (16.7)	4 (12.9)	5 (16.1)		3 (10.0)	6 (19.4)	5 (16.1)	
Others	15 (50.0)	16 (51.6)	10 (32.3)		17 (56.7)	17 (54.8)	7 (22.6)	
Duration of cirrhosis, (months), median (IQR)	30.0 (49.5)	12.0 (31.0)	6.0 (45.0)	0.154	16.5 (37.0)	36.0 (50.0)	9.0 (44.0)	0.341
Child–Pugh classification, *n* (%)								
A + B	26 (86.7)	28 (90.3)	31 (100.0)	0.093	28 (93.3)	29 (93.5)	28 (90.3)	1.00
C	4 (13.3)	3 (9.7)	0 (0.0)		2 (6.7)	2 (6.5)	3 (9.7)	
Hospitalization								
No	5 (16.7)	4 (12.9)	6 (19.4)	0.833	7 (23.3)	4 (12.9)	4 (12.9)	0.532
Yes	25 (83.3)	27 (87.1)	25 (80.6)		23 (76.7)	27 (87.1)	27 (87.1)	
Presence of comorbidities, *n* (%)				0.864				0.583
No	19 (63.3)	19 (61.3)	21 (67.7)		17 (56.7)	21 (67.7)	21 (67.7)	
Yes	11 (36.7)	12 (38.7)	10 (32.3)		13 (43.3)	10 (32.3)	10 (32.3)	
Prevalence of sarcopenia and its components								
Sarcopenia, *n* (%)	16 (53.3)	26 (83.9)	19 (61.3)	**0.032**	19 (63.3)	25 (80.6)	17 (54.8)	0.092
Low muscle strength^b^, *n* (%)	13 (43.3)	12 (38.7)	9 (29.0)	0.497	13 (43.3)	11 (35.5)	10 (32.3)	0.655
Low muscle function^c^, *n* (%)	24 (80.0)	31 (100.0)	25 (80.6)	**0.030**	27 (90.0)	27 (87.1)	26 (83.9)	0.925
Low muscle mass^d^, *n* (%)	14 (46.7)	14 (45.2)	20 (64.5)	0.238	14 (46.7)	20 (64.5)	14 (45.2)	0.260

^a^ Low levels of physical activity (PA): (PA) over the past 7 days < 600 (Met‐min/week), moderate levels of (PA): (600–3000 MET‐min/week) and high levels of (PA): ( > 3000 MET‐min/week) due to the low percentage (2.2%) in the high levels of (PA), we combined the two levels of moderate and high to moderate to high (PA).

^b^ Low muscle strength < 27 kg for females and for males, muscle strength < 16 kg for females.

^c^ A 6MWD of less than 205 m represents low muscle function.

^d^ Low muscle mass: upper arm circumference < 27.5 cm for females and for males upper arm circumference < 28.6 cm.

* Bolded values are significant at the < 0.05 level.

**Table 4:** Baseline characteristics of the study participants across categories of dietary pattern scores (cont.).

Dietary patterns	Healthy dietary pattern			*p* value	Plant‐based and light dietary pattern			*p* value*
	T1 (*n* = 30)	T2 (*n* = 31)	T3 (*n* = 31)		T1 (*n* = 30)	T2 (*n* = 31)	T3 (*n* = 31)	
Age (years)	47.40 ± 13.68	47.87 ± 14.60	51.97 ± 11.89	0.345	49.97 ± 12.64	44.84 ± 14.62	52.52 ± 12.20	0.072
Gender, *n* (%)								
Male	16 (53.3)	15 (48.4)	22 (71.0)	0.175	16 (53.3)	19 (61.3)	18 (58.1)	0.819
Female	14 (46.7)	16 (51.6)	9 (29.0)		14 (46.7)	16 (38.7)	113 (41.9)	
Occupation, *n* (%)								
Unemployed	8 (26.7)	11 (35.5)	8 (25.8)	0.493	9 (30.0)	9 (29.0)	9 (29.0)	0.983
Employee/retired	8 (26.7)	4 (12.9)	10 (32.3)		6 (20.0)	8 (25.8)	8 (25.3)	
Self‐employed	14 (46.7)	16 (51.6)	13 (41.9)		15 (50.0)	14 (45.2)	14 (45.2)	
Economic status, *n* (%)								
Poor	12 (40.0)	18 (58.1)	8 (25.8)	0.103	9 (30.0)	14 (45.2)	15 (48.4)	0.367
Moderate	16 (53.3)	11 (35.5)	18 (58.1)		17 (56.7)	13 (41.9)	15 (48.4)	
Good	2 (6.7)	2 (6.5)	5 (16.1)		4 (13.3)	4 (12.9)	1 (3.2)	
Marital status, *n* (%)								
Single/divorced/widowed	7 (23.3)	8 (25.8)	5 (16.1)	0.665	7 (23.3)	9 (29.0)	4 (12.9)	0.296
Married	23 (76.7)	23 (74.2)	26 (83.9)		23 (76.7)	22 (71.0)	27 (87.1)	
Education status, *n* (%)								
Illiterate	2 (6.7)	6 (19.4)	4 (12.9)	0.370	6 (20.0)	2 (6.5)	4 (12.9)	0.523
High school diploma or lower	25 (83.3)	20 (64.5)	20 (64.5)		20 (66.7)	22 (71.0)	23 (74.2)	
University degree	3 (10.0)	5 (16.1)	7 (22.6)		4 (13.3)	7 (22.6)	4 (12.9)	
Physical activity^a^ (MET)								
Low PA	23 (76.7)	25 (80.6)	24 (77.4)	0.951	20 (66.7)	27 (87.1)	25 (80.6)	0.143
Moderate to high PA	7 (23.3)	6 (19.4)	7 (22.6)		10 (33.3)	4 (12.9)	6 (19.4)	
Anthropometric indices								
Weight (kg)	69.42 ± 12.04	71.40 ± 13.77	75.97 ± 18.22	0.217	69.24 ± 14.06	73.09 ± 12.81	74.45 ± 17.73	0.378
Height (cm)	168.67 ± 11.32	170.32 ± 11.68	169.40 ± 16.40	0.889	168.20 ± 10.91	168.53 ± 17.13	171.65 ± 10.71	0.535
Body mass index (BMI)	24.46 ± 3.78	24.64 ± 3.98	25.32 ± 4.17	0.671	24.41 ± 4.09	25.01 ± 3.87	24.99 ± 4.00	0.8
Waist circumference (cm)	91.27 ± 11.96	91.79 ± 10.07	95.50 ± 13.91	0.330	91.18 ± 12.36	93.48 ± 11.54	93.89 ± 12.61	0.649
Arm circumference (cm)	27.33 ± 2.99	27.53 ± 3.69	28.45 ± 4.47	0.467	27.22 ± 3.55	28.56 ± 3.20	27.53 ± 4.40	0.344
Calf circumference (cm)	36.03 ± 7.78	34.86 ± 5.44	35.60 ± 4.49	0.747	35.00 ± 4.43	35.02 ± 4.78	36.43 ± 8.10	0.566
Etiology of cirrhosis, *n* (%)								
Alcoholic cirrhosis	3 (10.0)	2 (6.5)	4 (12.9)	0.745	3 (10.0)	1 (3.2)	5 (16.1)	0.689
Hepatitis	1 (3.3)	2 (6.5)	1 (3.2)		2 (6.7)	1 (3.2)	1 (3.2)	
Alcohol consumption	1 (3.3)	0 (0.0)	2 (6.5)		2 (6.7)	1 (3.2)	0 (0.0)	
Hepatitis B and C	2 (6.7)	3 (9.7)	2 (6.5)		1 (3.3)	4 (12.9)	2 (6.5)	
Autoimmune hepatitis	8 (26.7)	4 (12.9)	2 (6.5)		6 (20.0)	4 (12.9)	4 (12.9)	
Primary sclerosing cholangitis (PSC)	4 (13.3)	4 (12.9)	6 (19.4)		3 (10.0)	7 (22.6)	4 (12.9)	
Others	11 (36.7)	16 (51.6)	14 (45.2)		13 (43.3)	13 (41.9)	15 (48.4)	
Duration of cirrhosis, (months), median (IQR)	21.0 (58.5)	12.0 (43.0)	15.0 (31.0)	0.885	13.5 (30.2)	24.0 (55.0)	24.0 (56.0)	0.936
Child–Pugh classification, *n* (%)								
A + B	30 (100.0)	29 (93.5)	26 (83.9)	0.065	29 (96.7)	31 (100.0)	25 (80.6)	**0.010**
C	0 (0.0)	2 (6.5)	5 (16.1)		1 (3.3)	0 (0.0)	6 (19.4)	
Hospitalization								
No	7 (23.3)	4 (12.9)	4 (12.9)	0.532	6 (20.0)	4 (12.9)	5 (16.1)	0.732
Yes	23 (76.7)	27 (87.1)	27 (87.1)		24 (80.0)	27 (87.1)	26 (83.9)	
Presence of comorbidities, *n* (%)								
No	20 (66.7)	20 (64.5)	19 (61.3)	0.907	21 (70.0)	21 (67.7)	17 (54.8)	0.409
Yes	10 (33.3)	11 (35.5)	12 (38.7)		9 (30.0)	10 (32.3)	14 (45.2)	
Prevalence of sarcopenia and its components								
Sarcopenia, *n* (%)	21 (70.0)	26 (83.9)	14 (45.2)	**0.005**	21 (34.4)	20 (64.5)	20 (64.5)	0.873
Low muscle strength^b^, *n* (%)	11 (36.7)	11 (35.5)	12 (38.7)	0.965	9 (30.3)	10 (32.3)	15 (48.4)	0.265
Low muscle function^c^, *n* (%)	26 (86.7)	29 (93.5)	25 (80.6)	0.320	23 (76.7)	29 (93.5)	28 (90.3)	0.117
Low muscle mass^d^, *n* (%)	18 (60.0)	16 (51.6)	14 (45.2)	0.509	18 (60.0)	13 (41.9)	17 (54.8)	0.345

^a^ Low levels of physical activity (PA): (PA) over the past 7 days < 600 (Met‐min/week), moderate levels of (PA): (600–3000 MET‐min/week) and high levels of (PA): ( > 3000 MET‐min/week) due to the low percentage (2.2%) in the high levels of (PA), we combined the two levels of moderate and high to moderate to high (PA).

^b^ Low muscle strength < 27 kg for females and for males, muscle strength < 16 kg for females.

^c^ A 6MWD of less than 250 m represents low muscle function.

^d^ Low muscle mass: upper arm circumference < 27.5 cm for females and for males upper arm circumference < 28.6 cm.

* Bolded values are significant at the < 0.05 level.

Logistic regression analyses confirmed these associations (Tables 5 and 6). After adjusting for age, sex, energy intake, physical activity, waist circumference, alcohol intake, Child–Pugh classification and comorbidities (Model 2), adherence to the Western dietary pattern remained significantly associated with higher odds of sarcopenia in the second tertile (OR: 6.04; 95% CI: 1.53–23.93; *p* trend = 0.019). In contrast, the highest tertile of the Healthy dietary pattern was inversely associated with sarcopenia (OR: 0.38; 95% CI: 0.11–1.27; *p* trend = 0.022). No statistically significant associations were observed between the Unhealthy or Plant‐Based and Light patterns and sarcopenia after multivariable adjustments.

**Table 5:** Multivariable‐adjusted odds ratios (95% CIs) for sarcopenia and its components across tertile categories of dietary patterns.

Dietary patterns	Western dietary pattern OR (95% Cl)			*p* *_trend_	Unhealthy dietary pattern OR (95% Cl)			*p* *_trend_
	T1 (*n* = 30)	T2 (*n* = 31)	T3 (*n* = 31)		T1 (*n* = 30)	T2 (*n* = 31)	T3 (*n* = 31)	
Sarcopenia and its components								
Sarcopenia								
Crude	1	4.55 (1.37, 15.04)	1.38 (0.5, 3.83)	**0.041**	1	2.41 (0.75, 7.69)	0.70 (0.25, 1.96)	0.101
Model 1^a^	1	5.96 (1.63, 21.80)	1.61 (0.50, 5.18)	**0.023**	1	2.18 (0.64, 7.48)	0.46 (0.14, 1.52)	0.06
Model 2^b^	1	6.04 (1.53, 23.93)	1.04 (0.29, 3.69)	**0.019**	1	2.14 (0.58, 7.92)	0.36 (0.10, 1.30)	**0.038**
Low muscle strength^c^								
Crude	1	0.83 (0.30, 2.29)	0.25 (0.53, 1.54)	0.5	1	0.72 (0.26, 2.02)	0.62 (0.22, 1.77)	0.657
Model 1^a^	1	0.81 (0.28, 2.39)	0.62 (0.19, 1.99)	0.729	1	0.87 (0.29, 3.63)	0.97 (0.3, 3.16)	0.968
Model 2^b^	1	0.73 (0.24, 2.26)	0.55 (0.16, 1.89)	0.632	1	0.91 (0.29, 2.84)	0.86 (0.25, 2.98)	0.970
Low muscle function^d^								
Crude	1	7.50 (0.84, 66.61)	1.04 (0.29, 3.68)	0.174	1	0.75 (0.15, 3.67)	0.58 (0.12, 2.66)	0.779
Model 1^a^	1	8.03 (0.86, 74.75)	0.94 (0.23, 3.87)	0.148	1	0.67 (0.13, 3.49)	0.49 (0.09, 2.56)	0.701
Model 2^b^	1	7.95 (0.75, 84.44)	1.33 (0.27, 6.53)	0.219	1	0.66 (0.11, 3.96)	0.43 (0.07, 2.82)	0.682
Low muscle mass^e^								
Crude	1	0.94 (0.34, 2.58)	2.08 (0.74, 5.81)	0.244	1	2.08 (0.74, 5.81)	0.94 (0.34, 2.58)	0.244
Model 1^a^	1	0.99 (0.35, 2.80)	2.37 (0.78, 7.24)	0.199	1	1.99 (0.67, 5.86)	0.84 (0.29, 2.45)	0.262
Model 2^b^	1	0.78 (0.22, 2.73)	1.50 (0.41, 5.49)	0.608	1	2.53 (0.66, 9.64)	0.47 (0.13, 1.74)	0.065

^a^ Model 1: Adjusted for age, sex, and energy intake.

^b^ Model 2: Further adjusted for physical activity, WC, alcohol consumption, Child–Pugh classification and positive presence of comorbidities.

^c^ Low muscle strength < 27 kg for females and for males, muscle strength < 16 kg for females.

^d^ A 6MWD of less than 250 m represents low muscle function.

^e^ Low muscle mass: upper arm circumference < 27.5 cm for females and for males upper arm circumference < 28.6 cm.

* Bolded values are significant at the < 0.05 level.

**Table 6:** Multivariable‐adjusted odds ratios (95% CIs) for sarcopenia and its components across tertile categories of dietary patterns.

Dietary patterns	Healthy dietary pattern OR (95% Cl)			*p*_trend_	Plant‐based and light dietary pattern OR (95% Cl)			*p* *_trend_
	T1 (*n* = 30)	T2 (*n* = 31)	T3 (*n* = 31)		T1 (*n* = 30)	T2 (*n* = 31)	T3 (*n* = 31)	
Sarcopenia and its components								
Sarcopenia								
Crude	1	2.22 (0.64, 7.66)	0.35 (0.12, 1.01)	**0.007**	1	0.77 (0.26, 2.27)	0.77 (0.26, 2.27)	0.873
Model 1^a^	1	2.75 (0.90, 8.38)	5.79 (1.69, 19.79)	**0.016**	1	0.81 (0.26, 2.52)	0.87 (0.28, 2.63)	0.936
Model 2^b^	1	2.36 (0.64, 8.72)	0.38 (0.11, 1.27)	**0.022**	1	1.02 (0.29, 3.50)	1.16 (0.33, 3.99)	0.968
Low muscle strength^c^								
Crude	1	0.95 (0.33, 2.70)	0.39 (0.38, 3.07)	0.965	1	1.11 (0.37, 3.28)	2.18 (0.76, 6.26)	0.271
Model 1^a^	1	0.92 (0.30, 2.76)	0.95 (0.31, 2.88)	0.988	1	0.96 (0.31, 2.99)	1.94 (0.65, 5.77)	0.347
Model 2^b^	1	1.03 (0.34, 3.15)	1.05 (0.33, 3.36)	0.997	1	0.91 (0.27, 3.03)	1.78 (0.56, 5.65)	0.467
Low muscle function^d^								
Crude	1	2.23 (0.38, 13.20)	0.64 (0.16, 2.54)	0.347	1	4.41 (0.83, 23.30)	2.84 (0.65, 12.23)	0.141
Model 1^a^	1	1.63 (0.38, 6.94)	3.60 (0.63, 20.36)	0.347	1	4.62 (0.84, 25.41)	3.00 (0.68, 13.21)	0.130
Model 2^b^	1	2.10 (0.31, 14.26)	0.39 (0.08, 1.91)	0.202	1	3.34 (0.56, 19.99)	2.09 (0.44, 10.04)	0.353
Low muscle mass^e^								
Crude	1	0.71 (0.26, 1.96)	0.55 (0.19, 1.52)	0.511	1	0.48 (0.17, 1.33)	0.81 (0.29, 2.23)	0.350
Model 1^a^	1	1.74 (0.61, 5.00)	1.20 (0.43, 3.36)	0.570	1	0.45 (0.15, 1.30)	0.84 (0.30, 2.38)	0.301
Model 2^b^	1	0.76 (0.23, 2.52)	0.55 (0.15, 1.94)	0.649	1	0.75 (0.20, 2.74)	1.02 (0.29, 3.54)	0.874

^a^ Model 1: Adjusted for age, sex, and energy intake.

^b^ Model 2: Further adjusted for physical activity, WC, alcohol consumption, Child–Pugh classification and positive presence of comorbidities.

^c^ Low muscle strength < 27 kg for females and for males, muscle strength < 16 kg for females.

^d^ A 6MWD of less than 250 m represents low muscle function.

^e^ Low muscle mass: upper arm circumference < 27.5 cm for females and for males upper arm circumference < 28.6 cm.

* Bolded values are significant at the < 0.05 level.

While crude associations were observed between the Unhealthy dietary pattern and muscle mass loss, these did not remain significant after adjustment for confounders. Similarly, the Plant‐Based and Light pattern showed no consistent associations with sarcopenia or its components, although a significant relationship with Child–Pugh class was noted (*p* = 0.010), possibly indicating a role in disease severity rather than muscle outcomes.

## 4 Discussions

This study provides novel insights into the interplay between dietary patterns and sarcopenia among patients with liver cirrhosis, highlighting the critical role of nutrition in modulating muscle health within this vulnerable population. Our findings contribute to the growing body of evidence suggesting that diet quality, not merely energy or macronutrient adequacy, plays a pivotal role in preserving muscle mass and function in chronic liver disease (CLD) [35]. This highlights the importance of incorporating nutritional strategies into comprehensive management frameworks for cirrhosis.

We observed that over two‐thirds of the study population met diagnostic criteria for sarcopenia or severe sarcopenia, consistent with previous studies indicating a high prevalence of muscle depletion among cirrhotic patients [3, 36]. Importantly, key anthropometric and functional indicators, BMI, limb circumferences, muscle strength, and physical performance, were markedly reduced in sarcopenic individuals. These physical impairments reflect the profound catabolic state driven by both the underlying liver disease and dietary insufficiencies [37, 38].

Dietary pattern analysis revealed significant associations between habitual food intake and the risk of sarcopenia. Notably, moderate adherence to a Western dietary pattern, characterized by high consumption of red and processed meats, organ meats, and refined carbohydrates, was significantly associated with elevated odds of sarcopenia, even after adjusting for confounders such as age, physical activity, and comorbidities. These results align with the existing literature, which links Western‐style diets to systemic inflammation, insulin resistance, and adverse metabolic outcomes, all of which may contribute to muscle catabolism in cirrhosis [39, 40].

Conversely, adherence to a Healthy dietary pattern rich in vegetables, nuts, liquid oils, and whole grains was inversely associated with sarcopenia [7]. Participants in the highest tertile of this pattern demonstrated significantly lower odds of muscle loss, suggesting a protective effect possibly mediated by higher antioxidant intake, improved micronutrient status, and anti‐inflammatory dietary components [40, 41]. These findings echo the growing recognition of the Mediterranean and DASH diets as beneficial for preserving muscle integrity and physical function in ageing and chronically ill populations [42]. Notably, no significant associations were found for the Unhealthy or Plant‐Based and Light patterns after adjustment, suggesting the complexity of plant‐forward diets, which may not uniformly confer benefits in the absence of nutrient diversity or adequate protein intake.

An unexpected yet noteworthy observation was the association between the Plant‐Based and Light dietary pattern and the severity of advanced liver disease, as reflected in the Child–Pugh classification. While this pattern showed no consistent relationship with sarcopenia, its association with disease stage may suggest that patients with more advanced cirrhosis shift toward lighter, less protein‐rich diets, potentially reflecting self‐imposed dietary restrictions or reduced appetite. This highlights the clinical challenge of balancing protein adequacy with disease tolerance in the management of cirrhosis [43, 44].

### 4.1 Clinical Implications and Translational Relevance

These results carry important clinical implications. First, dietary assessment should become an integral component of sarcopenia screening in patients with liver disease. Second, patient education on the benefits of high‐quality, nutrient‐dense diets may offer a non‐pharmacological strategy to mitigate sarcopenia progression. Third, the findings support the development of culturally tailored nutrition interventions, especially in Middle Eastern populations where dietary practices may diverge from Western norms.

Given the high burden of sarcopenia in cirrhosis and its impact on quality of life, transplant outcomes, and mortality [45, 46], the integration of dietary pattern evaluation into hepatology care pathways is warranted. Future clinical trials should test whether dietary modification, possibly in combination with physical activity and amino acid supplementation, can reverse or prevent muscle wasting in this context.

### 4.2 Strengths and Limitations

Key strengths of this study include the detailed and culturally adapted 168‐item food frequency questionnaire (FFQ), the use of validated diagnostic criteria for sarcopenia, and the comprehensive multivariable adjustment for potential confounders. To our knowledge, this is one of the few studies in a Middle Eastern population to investigate the relationship between empirically derived dietary patterns and sarcopenia in cirrhosis, thereby addressing a significant geographic and epidemiological gap.

Nevertheless, several limitations merit consideration. First, the cross‐sectional design precludes causal inference, and reverse causation cannot be ruled out; for example, individuals with sarcopenia may adopt specific dietary habits as a result of their disease state. Second, dietary intake was self‐reported and subject to recall bias, although validated tools were employed. Third, muscle mass was evaluated using mid‐arm muscle circumference, a practical and validated field method, yet less precise than imaging‐based or bioelectrical impedance analysis (BIA) techniques. Fourth, the modest sample size may have limited the power to detect associations with less common patterns or stratified outcomes. Finally, we did not assess circulating biomarkers of inflammation or nutrient status, which could have provided mechanistic insights.

Despite these limitations, this study provides novel regional and analytical insights, establishing a methodological foundation for future large‐scale and longitudinal investigations that explore diet‐sarcopenia relationships in liver cirrhosis.

## 5 Conclusion

In conclusion, our findings highlight the crucial role contribution of dietary quality in maintaining muscle health in patients with liver cirrhosis. While Western‐style diets are associated with greater risk of sarcopenia, adherence to healthy dietary patterns may offer a modifiable protective factor. These results reinforce the imperative for nutritional counseling and tailored interventions in the routine care of cirrhotic patients.

## Author Contributions

**Elham Sobhrakhshankhah:** writing – original draft, writing – review and editing, investigation, conceptualization, validation, data curation, methodology, resources, supervision, and project administration. **Parvin Hassanzadeh:** writing – original draft, writing – review and editing, formal analysis, data curation, investigation, resources, validation, and visualization. **Soghra Karimi:** data curation, methodology, writing – review and editing, and validation. **Mohsen Reza Mansoorian:** writing – original draft, writing – review and editing, data curation, investigation, resources, validation, supervision, and project administration. **Naemeh Kolahdoozan:** writing – original draft, writing – review and editing, data curation, investigation, resources, validation, and visualization. **Azam Doustmohammadian:** conceptualization, data curation, supervision, methodology, software, investigation, funding acquisition, writing – original draft, writing – review and editing, formal analysis, validation, visualization, project administration, and resources.

## Conflicts of Interest

The authors declare no conflicts of interest.

## Transparency Statement

The corresponding author, Azam Doustmohammadian, confirms that this manuscript offers a truthful, complete, and transparent account of the research conducted. No critical elements have been excluded, and any deviations from the original study plan or protocol have been clearly stated.

## Supporting information


**Figure S1:** Scree plots of the eigenvalues to determine the appropriate number of dietary patterns. *Note:* Each point represents an extracted component (dietary patterns), and the curve indicates the decline in eigenvalues across components. The “elbow” point, where the slope levels off, suggests the optimal number of components to retain for further analysis.

**Table S1:** Components of food groups included in dietary pattern.

## Data Availability

The data sets generated and analysed during the present study can be made available by the corresponding author, Dr Azam Doustmohammadian (doost_mohammadi@yahoo.com), upon reasonable request.
