# Supplementary material for: Association Between Dietary Patterns and Sarcopenia in Patients With Liver Cirrhosis: A Cross‐Sectional Study
Source: Health Sci Rep. 2026 Jun 11;9(6):e72610. doi: 10.1002/hsr2.72610 (PMC13259968; doi:10.1002/hsr2.72610)
Supplement: Supplementary file 1 — Figure S1: Scree plots of the eigenvalues to determine the appropriate number of dietary patterns. Note: Each point represents an extracted component (dietary patterns), and the curve indicates the decline in eigenvalues across components. The “elbow” point, where the slope levels off, suggests the optimal number of components to retain for further analysis. [file HSR2-9-e72610-s001.docx]

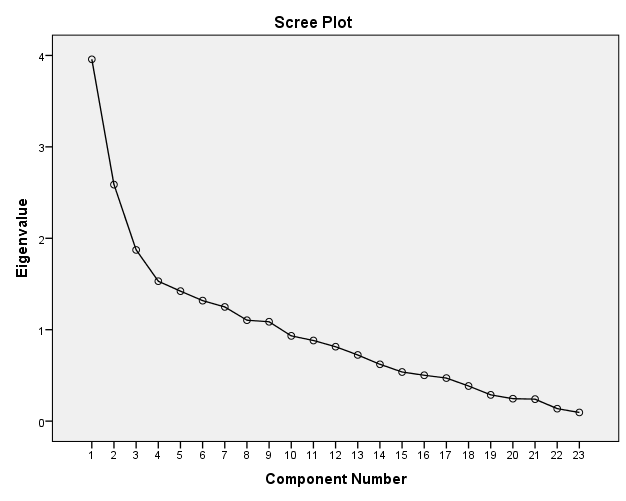


**SUPPLEMENTARY FIGURE 1. Scree plots of the eigenvalues to determine the appropriate number of dietary patterns**

Note: Each point represents an extracted component (dietary patterns), and the curve indicates the decline in eigenvalues across components. The “elbow” point, where the slope levels off, suggests the optimal number of components to retain for further analysis.
