# Supplementary material for: Association Between Dietary Patterns and Sarcopenia in Patients With Liver Cirrhosis: A Cross‐Sectional Study
Source: Health Sci Rep. 2026 Jun 11;9(6):e72610. doi: 10.1002/hsr2.72610 (PMC13259968; doi:10.1002/hsr2.72610)
Supplement: Supplementary file 2 — Table S1: Components of food groups included in dietary pattern. [file HSR2-9-e72610-s002.docx]

**TABLE S1 Components of food groups included in dietary pattern**

| Foods or food groups | Food items |
| --- | --- |
| Whole grains | Brown bread; Barely; Corn |
| Refined grains | White bread; Crispbreads; Spaghetti; Cooked rice; Low-sugar dry biscuits |
| Potatoes | Boiled potato; Cooked potato |
| Fried potatoes | Fried potatoes |
| Processed meats | Sausage; [kielbasa](https://abadis.ir/entofa/kielbasa/); Pizza; Hamburger |
| Red meat | Beef meat & Lamb meat (tournedos, steak, mince); |
| Organ meats | Organs meats of lamb and/or beef: liver, brain, tongue |
| White meats | Fish; poultry; canned tuna |
| Eggs | Eggs |
| Legumes | Lentils; Beans; Peas; Chickpeas; Soybeans |
| Nuts | Nuts and seeds |
| Vegetable oils | Olive oil; Sesame oil; Canola or Sunflower oil; Vegetable hydrogenated oil |
| Solid fats | Solid fat; Animal oils; Cream; Butter |
| Dairy products (low-fat) | Plain semi-skimmed milk; Plain skimmed milk; Low-fat yogurts; Low-fat cheeses; Low-fat dairy drinks (dough) |
| Dairy products (full-fat) | Plain whole milk; Flavored milk; Natural whole yogurts; Soft rind cheeses; full-fat white cheeses |
| Fruits | All types of fresh fruits, dried fruits, dates, fresh fruit juices |
| Vegetables | All types of fresh, cooked, and dried vegetables as well as vegetable juice |
| Salty & Sweet snacks | Salty biscuits; Crisps; Crackers; Cookies; Brown or white sugar; Sweets; Cakes; Honey; Jam; Candy; Halva; Chocolates; Candied fruit |
| Soft drinks | Carbonated Sugar-sweetened soft drinks; Industrial fruit juices |
| High-fat/high sodium condiments | Mayonnaise; ketchup; Sour and salty pickles; |
| Spices | Various spices (ginger, turmeric, black and red pepper, cinnamon) |
| Tea & Coffee | Tea; Coffee |
| Salt | Salt |
